# Zn Fertilizer and Mycorrhizal Inoculation Effect on Bread Wheat Cultivar Grown under Water Deficit

**DOI:** 10.3390/life13051078

**Published:** 2023-04-24

**Authors:** Neila Abdi, Angeline Van Biljon, Chrisna Steyn, Maryke Labuschagne

**Affiliations:** Department of Plant Sciences (Plant Breeding), University of the Free State, Bloemfontein 9300, South Africa

**Keywords:** bread wheat, AMF, zinc, drought, growth parameters, osmolyte, osmoprotector, ionic attributes

## Abstract

During drought stress, many enzymes are inactivated in plants due to Zn deficiency. Zn application and arbuscular mycorrhiza fungi (AMF)–wheat symbiosis reportedly improve the tolerance of plants to drought stress. This study was done to investigate the effect of Zn and AMF on plant growth, yield attributes, relative water content (RWC), harvest index (HI), photosynthetic activity, solute accumulation, glycine betaine (GB) accumulation, antioxidant activities [(catalase (CAT) and superoxide dismutase (SOD)], and ionic attributes in a bread wheat cultivar (SST806) under drought-stress in plants grown under greenhouse conditions. Zn application and AMF inoculation, separately and combined, enhanced all plant growth parameters and yield. Root dry weight (RDW) was increased by 25, 30, and 46% for these three treatments, respectively, under drought conditions compared to the control treatment. Overall, Zn application, AMF inoculation, and their combination increased protein content, RWC, and harvest index (HI) under drought stress. However, AMF inoculation improved proline content more than Zn application under the same conditions. Regarding GB accumulation, AMF, Zn, and the combination of Zn and AMF increased GB under drought compared to well-watered conditions by 31.71, 10.36, and 70.70%, respectively. For the antioxidant defense, AMF inoculation and Zn application improved SOD and CAT activity by 58 and 56%, respectively. This study showed that Zn and/or AMF increased antioxidant levels and ionic attributes under abiotic stress.

## 1. Introduction

Abiotic stresses negatively affect crop production [1]. Water deficit is known to decrease plant growth, significantly reducing yield [2]. Less water is considered a key climatic problem that directly decreases crop production, such as cereals, globally [3]. Drought stress causes severe losses in wheat yield in different growing regions worldwide. As the largest contributor to total consumed calories by humans, wheat represents the principal dietary staple in the world [4]. Yield and its attributes are highly affected by drought in the different stages of the growing cycle of plants [5]. Drought stress decreased wheat yield by as much as 60% [6]. As a strategy for drought tolerance improvement, crops escape water deficit, especially in the climate change conditions currently being experienced [7]. Several physiological and biochemical alterations are induced by drought, causing plants to have many adaptation strategies as defensive survival mechanisms against drought stress. It was reported that different strategies could be followed to reduce food production decreases due to drought in the future [8,9]. The development of drought tolerance mechanisms in food crops is one such strategy. Plants have many options to escape drought stress effects, such as water uptake and flow in plant tissues, production of osmolytes and antioxidant activities, and photosynthesis mechanisms [10]. Moreover, plants were found to produce more osmolyte and soluble sugars and have increased antioxidant defense mechanisms (such as SOD and CAT) to combat the toxic effects of the overproduction of reactive oxygen species (ROS) [11]. Due to the water deficit, genes encoding antioxidant enzymes were activated in tolerant genotypes. The wheat genome was known for some modification in terms of genes to control drought-stress conditions [12].

Zn fertilizer and AMF inoculation can contribute to plant survival and tolerance of water deficit conditions in many crops, such as wheat [13]. Zn application maintained nutrient balance and stomata reaction in different crops to reduce the effects of drought stress [14]. As an antioxidant reaction, SOD and CAT enzymes were enhanced due to Zn fertilization in response to water deficit.

Zn is classified as a necessary micronutrient for plant growth due to its involvement in carbon metabolism [15]. Zn plays an important role in plant nucleic acid metabolism. Several biomolecules as lipids and proteins, contain Zn as an essential component; also, it is a cofactor for many enzymes [15,16]. Many studies showed the plant responses to Zn application [17]. Physiological and biochemical processes such as plasma membrane functions and oxidative stress tolerance depend on Zn content [18].

Zn application also reduced the alteration of membrane permeability and the damage caused by oxidative and peroxidative reactions [19,20]. An adequate supply of Zn can reduce the effects of drought on different crops, such as wheat [21].

Many reports confirmed that Zn plays an important role as a strategic component for the root and shoot system and a cofactor of many enzymes [22]. Yield attributes of wheat were increased due to Zn application [23,24]. Soil Zn application increased grain yield by 29%, whole-grain Zn concentration by 95%, and whole-grain estimated Zn bioavailability by 74% [25].

Arbuscular mycorrhizal fungi (AMF) belong to the phylum Glomeromycota, are soil inhabitants, and can colonize 80% of the roots. Mycorrhizal characteristics are mutually beneficial. AMF provide the host plant with essential nutrients (especially P) and water, and photosynthates are transported into endosymbiotic AMF for its development. Mycorrhizal mycelium feeds plants with several secondary metabolites and carbohydrates. It also improves plants to fix nitrogen and increase osmotic adjustments during water deficit.

The effect of AMF colonization depends on the host-plant interaction [26]. To tolerate drought stress, for example, in wheat, symbiosis with AMF can increase plant tolerance against this stress [27,28]. Antioxidant reaction, osmotic adjustments, and root hydraulic conductivity are better regulated in AM-plant association [29]. Zn uptake by the plant increased in the presence of AMF; however, the assimilation depends on the crop–AMF symbiosis. Mycorrhizal association contributed to Zn uptake of up to 24.3% of the total aboveground Zn in wheat and up to 12% in barley. At low Zn application, the highest contribution by the mycorrhizal pathway was observed in barley. Besides this, the grain yield of bread wheat was increased by AMF [30]. The use of Zn and AMF as fertilizer is one of the most effective strategies that can reduce the effect of drought stress and improve yield and plant growth. In addition, the use of biocontrol and chemical fertilizers was increased to reduce the impact of stress factors on crops. Moreover, AMF colonization could improve the nutrient uptake of a crop such as wheat in different types of soil by enhancing the root surface absorption area [31]. In the case of soil containing heavy metals, it was reported that mycorrhizal colonization could reduce the uptake of these metals [32]. Many studies investigated the role of AMF under drought stress to improve plant nutrient uptake. The synergistic interaction of AMF and Zn could improve concentrations of different micronutrients. It was reported that AMF with extraradical mycelium in the soil improved immobile nutrient (such as P and Zn) uptake by the host plant [33], causing an increase in the exchange of photosynthesis products from the plant to the fungus.

Glycine betaine accumulation works as an osmolyte in protecting organisms against abiotic stresses via osmoregulation or osmoprotection. As an osmoregulator, GB enhances root water assimilation, reduces the damage caused by oxidative reactions, and increases drought tolerance [34]. Due to the Zn application, compatible solutes were increased under drought stress [35]. GB maintains water retention in plants owing to Zn application that increases chlorophyll content and plant dry weight [36,37]. GB accumulation helpsplants to overcome drought and saline stress conditions. For example, in transgenic apples expressing the stress regulator gene, *Osmyb4*, accumulation of GB was linked to increased tolerance under drought and cold stress [38]. In chloroplast stroma, GB is produced by betaine aldehyde dehydrogenase (BADH). Under abiotic stress such as salinity, the enzyme choline monooxygenase (CMO) converts choline into betaine aldehyde and then an NAD+-dependent enzyme to improve tolerance against this stress [39].

To determine the effects of Zn application and/or AMF inoculation on bread wheat under drought stress, the regulation of various antioxidants, metabolites, and morphological traits was studied. It was hypothesized that Zn and AMF could improve bread wheat production under water deficit conditions.

## 2. Materials and Methods

### 2.1. Biological Materials and Growth Conditions

Seeds of one commercial South African wheat cultivar (SST806, official standard for spring wheat quality) were planted in plastic pots containing 2 kg of soil collected from 1.5 m deep subsoil (Table 1). They were grown under glasshouse conditions at the University of Free State, Bloemfontein, South Africa, from May 2019, with day/night temperatures of 18 °C at night and 22–24 °C during the day. The relative humidity during the day and night was 78%. A soil meter (Efekto Ltd., Caledon, South Africa) was used in this study. A completely randomized block designwas replicated three times for each treatment;control (T0), Zn (T1) = 40 kg ha^−1^, Arbuscular mycorrhizal fungi = AMF (T2), drought stress (T3), Zn+AM (T4), and Zn+AM+drought (T5).

### 2.2. Growing Conditions

Drought stress was applied at the three-leaf stage. When soil water content reached 25% field capacity, plants were allowed to receive water again; however, the well-watered conditions represented 100% field capacity. Before rewatering plants, a soil meter was used to measure soil water content.

### 2.3. Plant Biomass

Different plant samples (roots, shoots, and seeds) were dried until they attained a constant weight following the method previously described [40]. Plants were separated at 80 days after sowing (DAS) in root and shoots for various physiological and biochemical analyses.

### 2.4. Chlorophyll Content

Chlorophyll extraction was carried out from leaf discs of plants following the method previously described [41], and chlorophyll a, b, and total chlorophyll were computed from the extinction values following the equation of [42].

### 2.5. Total Protein, Relative Water Content, and Harvest Index

Total protein was estimated following the method previously described by Bates et al. [43]. Leaf relative water content (RWC) was calculated by the method described by Grieve & Grattan [44]. For chlorophyll a and b extraction, leaf discs of plants were mixed with 5 mL of 80% acetone overnight. After centrifugation, the supernatant was used for absorbance reading at 645 nm (chl a) and 663 nm (chl b) using a spectrophotometer (Hitachi-U2001, Tokyo, Japan). Relative water content (RWC) was measured following Cavell [45], where selected leaves were rehydrated by soaking in deionized water for 24 h. Fully turgid leaves were weighed and, subsequently, oven-dried for 48 h at 80 °C. Here, FW is fresh weight, DW is dry weight, and TW is turgid weight. Plant yield efficiency in terms of the harvest index (HI) was computed according to Mehraban et al. [46]. The amount of aboveground biomass production invested into harvestable organs was calculated as follows:

HI = (Seed dry weight/Aboveground plant biomass at harvest) × 100

### 2.6. Proline and Glycine Betaine Content

Proline content was analyzed following absorbance of toluene soluble brick-red colored complex at 520 nm [47]. The concentration of proline was estimated by referring to a standard curve drawn from known concentrations of proline. GB was determined following the absorbance of the betaine–peridotite complex with iodide in an acidic medium at 360 nm as per the method of Dubois et al. [48]. Reference standards of GB were prepared as 50–200 µg mL^−1^ for sample estimation.

### 2.7. Catalase and Superoxide Dismutase Estimation

CAT and SOD were measured using 0.2 g fresh leaf samples. The obtained mixture (0.05 M Tris–HCl buffer (pH = 7.5) and samples) was centrifuged at 13,000 rpm for 20 min at 4 °C.

After centrifugation, the supernatant was used to estimate CAT according to a modified method of Kar and Mishra [49], and SOD was assayed by the method described by Beauchamp and Fridovich [50].

### 2.8. Nutrient Analysis and Zinc Content

Nutrient extraction was done according to Carvalho et al. ([51], modified). Two g of flour for each sample was placed in labeled crucibles and ashed for 3 h in a furnace at 550 °C. Samples were digested with 2–2.5 mL of concentrated HNO_3_,then placed into the furnace at 550 °C for 1h. After that, 10 mL of diluted HNO_3_ (HNO_3_:H_2_O 1:2 dilution ratio) was added to the sample and placed for 5 min in a sand bath. The mixture was filtered through Whatman paper for purification. The atomic absorption spectroscopy (AAS) (Varian AAS FS 240 Model, Varian Inc., Palo Alto, CA, USA) method was used to analyze the mineral concentration. Five replicates were done per sample.

### 2.9. Statistical Analysis

Each parameter was investigated in its separate independent experiment. Analysis using variance (ANOVA) was performed, and subsequent comparison of the means was done using Duncan’s multiple range test at *p* = 0.05. Treatment mean *±* SE (*n* = 12) are for growth and yield attributes and (*n* = 4) for the other tested characteristics.

## 3. Results

### 3.1. Plant Growth, Yield, and Yield-Related Traits

Drought stress significantly affected (*p* < 0.05) growth parameters, yield, and yield components (Table 2). Zn application and AMF inoculation significantly enhanced plant growth and yield components under well-watered conditions and drought stress. Zn application and/or AMF inoculation enhanced all growth parameters and yield attributes. For example, RDW increased by 25, 30, and 46%, respectively, for these three treatments, compared to the control treatment. For 1000-grain weight, the increase was 9, 0.4, and 3% for the same three treatments (Table 2). Drought stress significantly decreased plant growth and grain yield attributes. The combination of Zn application and AMF inoculation alleviated the adverse effect of drought stress on all parameters except for grain weight per spike, which decreased by 45.9%. The decrease in 1000-grain weight was noticeably smaller after the application of Zn and AMF compared to drought stress only (Table 2). Under this constraint, Zn significantly enhanced 1000-grain weight. However, AMF or Zn did not affect grain number and grain weight per spike under drought stress (Table 2).

### 3.2. Chlorophyll Content

**Figure 1:**
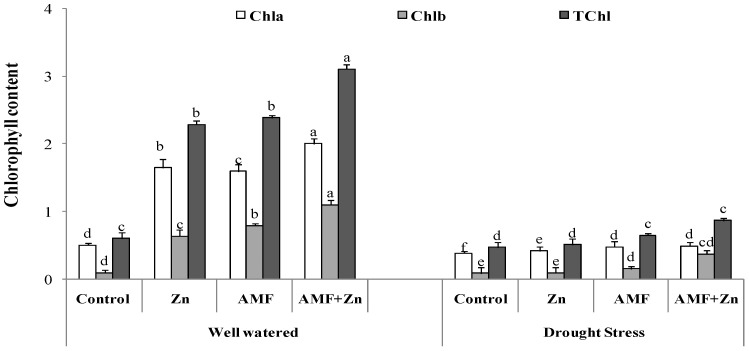
Individual and combined application of Zn and/or AMF effects on chlorophyll a, chlorophyll b, and chlorophyll (a+b) of a bread wheat cultivar under control (well-watered) and drought- stress conditions. Bars with different letters are significantly different at *p* ≤ 0.05.

There was significant variability of chlorophyll content due to Zn fertilization and AMF inoculation. Chlorophyll compounds were increased by Zn and AMF inoculation and their combination under both control and drought conditions. Chl a content increased by 69, 68, and 75%, Chl b content by 84, 87, and 90%, and Chl a+b content by 73, 74, and 80%, respectively, after the application of Zn and AMF inoculation and their combination compared with the control. However, there were nonsignificant effects on Chl a/b content under drought stress after Zn and AMF treatments. Overall, the highest chlorophyll content was observed in the plants treated with combined Zn and AMF under both control and drought-stress conditions (Figure 1).

### 3.3. Protein, Relative Water Content, and Harvest Index

**Figure 2:**
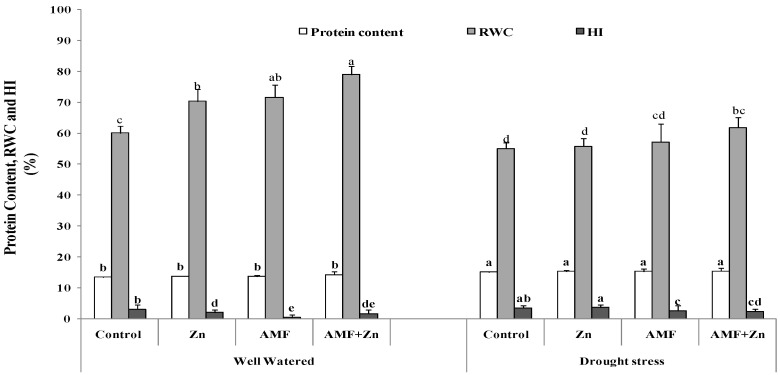
Zn application and/or AMF inoculation effect on protein content, relative water content (RWC), and harvest index (HI) of bread wheat cultivar under control (well-watered) and drought- stress conditions. Bars with different letters are significantly different at (*p* < 0.05).

Protein, relative water content, and HI were significantly(*p* ≤ 0.05) affected by drought stress. However, AMF inoculation and/or Zn and their combination improved protein content by about 15%. The highest level was 15.35% in plants that received combined Zn and AMF treatment under drought stress (Figure 2). Under well-watered conditions, Zn application, AMF inoculation, and their combination enhanced RWC by 14.10, 16.23, and 23.90%, respectively (Figure 2), although it decreased by 20.35, 20.15 and 21.66%, respectively, under drought stress. Under drought stress, Zn application and/or AMF inoculation enhanced HI by 45.91, 84.80 and 28.82%, respectively, compared to control conditions (Figure 2).

### 3.4. Accumulation of Glycine Betaine and Proline Content under Drought Stress

**Figure 3:**
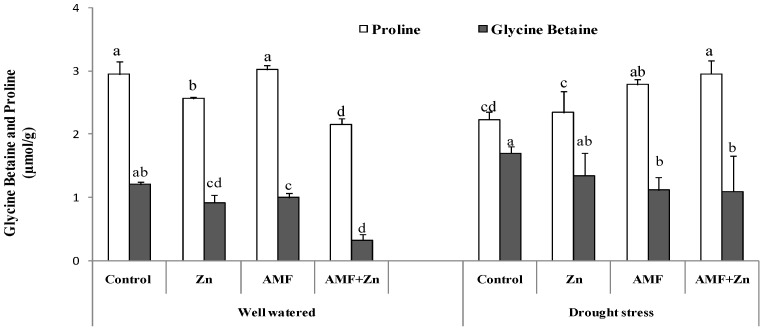
Zn application and/or AMF inoculation effect on glycine betaine and proline content in the bread wheat under control (well-watered) and drought-stress conditions. Bars with different letters are significantly different at *p* ≤ 0.05.

Treatment effects were significant for GB and proline. Under drought stress, the application of Zn and/or AMF inoculation increased GB compared to control conditions by 31.71, 10.36, and 70.70%, respectively. However, the level of GB was higher in the control under the same conditions (1.69 µmol g^−1^). Regarding proline content, results showed significant variability (*p* < 0.05) under both control and drought conditions. AMF inoculation improved proline content more than Zn application. Generally, drought stress decreased proline content compared to control conditions (Figure 3).

### 3.5. Activities of Antioxidant Enzymes

**Figure 4:**
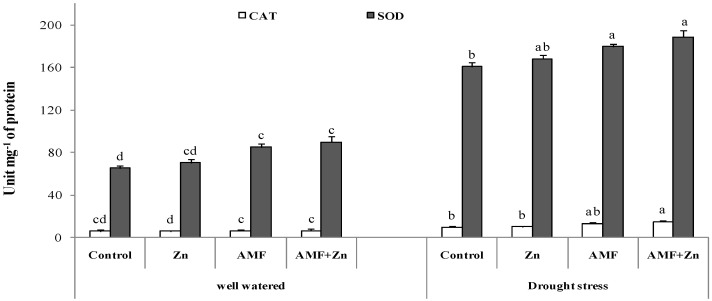
Zn application and/or AMF inoculation effects on catalase (CAT) and peroxide dismutase (SOD) in the bread wheat under control (well-watered) and drought-stress conditions. Bars with different letters are significantly different at *p* ≤ 0.05.

The antioxidant defense was enhanced significantly (*p* < 0.05) under drought stress mostly for peroxide dismutase activity, and the increase was outworn by 50% for all the treatments compared to the well-watered conditions. AMF inoculation and Zn application improved SOD and CAT activity by 58 and 56%, respectively, under drought stress (Figure 4). Under well-watered conditions, Zn and/or AMF did not significantly a meliorate the enzymatic reaction (Figure 4).

### 3.6. Nutrient Composition of Wheat Flour

**Figure 5:**
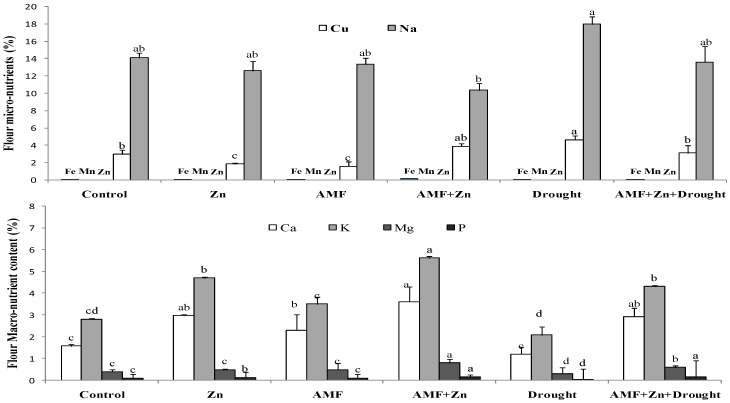
Zn application and/or AMF inoculation effect on macronutrient and micronutrient content in bread wheat cultivar under control (well-watered) and drought-stress conditions. Bars with different letters are significantly different at *p* ≤ 0.05.

Macro and/or microelements in wheat flour showed significant variability due to the combination of Zn application and AMF inoculation under drought stress (Figure 5). However, treatment effects were nonsignificant under control conditions for micronutrients. Drought stress significantly increased Na and Cu, compared to the control, by 21.68 and 36.13%, respectively. The microelements Fe, Mn, Zn, and Cu in the flour had very low concentrations (0.003–0.089%). On the contrary, macro elements were significantly affected by drought stress. Zn and/or AMF inoculation improved K, Ca, and P. For example, Zn combined with AMF increased K and P by 51.61 and 75%, respectively, under drought stress (Figure 5).

## 4. Discussion

The main objective of this study was to analyze the potential of Zn fertilizer and AMF for improving wheat performance under drought stress. Drought significantly affects wheat yield worldwide [52,53]. AMF improved water assimilation in many plants under drought stress as fungus mycelia can penetrate the soil and increase water absorption and transportation from roots to other plant parts as a tolerance mechanism to drought stress [54]. Fertilization using several nutrient sources increased plant vigor against environmental stress [55]. Zn fertilization and its co-application with AMF were evaluated by studying variability in different physiochemical mechanisms as described in a previous study [30]. AM fungus and/or Zn application positively affected morphological traits, increasing plant growth and yield attributes, as was reported previously [3]. Therefore, the efficiency of Zn and AMF application is confirmed in this study. Drought stress decreased plant dry weight and length. This was confirmed in another study [56]. Osmotic variability due to variations in osmotic potential caused a significant decrease in the fresh weight of plants due to a decrease in cellular division, consequently causing a decrease in total plant weight [57]. Zn combined with AMF treatment effectively improved plant growth under drought stress by sustaining higher water content in cells, thus ameliorating drought stress. All parameters were alleviated by Zn application and AMF inoculation, except for grain weight per spike, which decreased by 45.9% under drought stress. Zn improved chlorophyll synthesis, as it acts as a catalyst and cofactor of various enzymes [58]. This finding was confirmed in this study. Cell membranes, which cause improvement in the photosynthetic process, were protected by the application of Zn and AMF [59]. Similar findings were observed in rice and wheat plants. Zn increased all studied photosynthetic pigments [60].

Protein content was significantly enhanced only under stress conditions, and the effect was increased with Zn treatment. The potential effect of Zn on soluble protein in wheat under drought stress was previously reported [61]. Also, amino acid synthesis, which helps in protecting plants from drought stress, is related to Zn application [62]. Faced with drought stress, plant tolerance can be improved via drought escape by early flowering time in drier environments, avoidance by transpiration regulation, development of extensive root systems, trait flexibility, maintenance of water management in tissues, antioxidant scavenging, and secretion of plant growth substances by plant growth regulators and osmotic regulation [63]. Under drought stress, plants used stomatal closure to reduce the transpiration rate, causing an increase in leaf temperature. However, compared to the control, under the same conditions, Zn and/or AMF increased RWC and HI. These findings confirmed that Zn, at an optimum dose, maintained water status, stomatal conductance, and osmotic adjustment in many plants, such as chickpea, under drought stress [64]. For osmotic homeostasis regulation under stress conditions, proline as an osmolyte played an important role in protecting plants against drought [65]. The compatible solute accumulation leads to improved turgor potential and water content of plants, which contributes to enhanced plant growth performance under stress conditions. AMF was also reported to stimulate compatible solute and protein content under stress conditions [66]. The results of this study confirmed previous findings [67], mentioning that Zn and AMF acted synergistically to enhance proline and total protein content.

Drought stress reduces the assimilation of nutrients and inhibits the activities of important enzymes that are involved in the synthetic processes of energy for plant growth. For that, plants have an antioxidant defense against stress conditions, having different antioxidant reactions protecting plants under water deficit [68].This defense reaction was expressed by different enzymes which convert these harmful oxygen species to reduce their negative effect on plant growth [69]. In our study, drought stress increased levels of CAT and SOD compared to the control (well-watered). AMF inoculation or Zn application enhanced the activity of these antioxidant enzymes under drought-stress conditions, being more pronounced when applied together. This finding was confirmed by many reports mentioning enhancement in the enzymatic antioxidant defense system due to AMF and/or Zn application in wheat under drought stress [70]. Zn reduced oxidative damage under stress conditions, which confers stress tolerance to plants [71]. The increase in antioxidant enzyme activity is assessed through decreased malondialdehyde content and H_2_O_2_ content as noted in many crops, for example, in sunflower (*Helianthus annuus*), chickpea (*Cicer arietinum*) [72], lentil (*Lens culinaris*) [73], and wheat leaves.

Moreover, nonenzymatic molecules, such as the accumulation of GB in wheat leaves, decreased the impact of drought stress as an antioxidant defense. Zn and AMF increased the activity of GB under drought stress, being more pronounced when applied together (Figure 3). It was reported [74] that enzymatic antioxidant defense systems were enhanced in wheat due to Zn application under drought-stress conditions.

In addition, as a strategy to tolerate stress, plants balance the concentrations of macro and micro elements. The results showed that drought stress significantly increased Na and Cu. However, Fe, Mn, Zn, and Cu were present in the flour at very low concentrations (0.003–0.089%). Many reports confirmed this finding explaining that a different nutrient supply as Zn and biofertilizer (AMF) can increase plant growth under water stress, depending on the severity of the drought, the concentration of the elements in the soil, and other conditions [75]. Application of Zn, AMF inoculation, and their combination increased K, Ca, Mg, and P. For example, Zn combined with AMF increased K and P by 51.61 and 75%, respectively, under drought stress.

## 5. Conclusions

This study investigated the effect of Zn application and AM fertilization. Wheat growth, yield, the antioxidant mechanism (enzymes, osmoprotectors, and osmolytes), and nutrient balance were improved. Root proliferation was significantly enhanced due to Zn and AMF fertilization under stress conditions. Zn fertilizer combined with AMF had larger impacts on measured traits. As a work perspective, deep research is needed to be done under field conditions to confirm these results on the effects of Zn and AMF and to be recommended to improve wheat production under drought stress. Moreover, extensive work on molecular studies as the contribution of differentially expressing endogenous genes encoding antioxidant enzymes should be established.

## Figures and Tables

**Table 1:** Soil, Zn, and AMF characteristics used in the trial.

Soil		AMF Characteristics and Zinc Application
pH	6.8	- 150 g per 150 kg of seed was applied
Sand	50%	
Silt	10%	- It is a commercial inoculum in powder form, registered and produced by Biocult (Pty) Ltd. 005333/07, Somerset West, South Africa
Clay	40%	
Phosphorus (P)	7.5 mg kg^−1^	- Active ingredient was mycorrhizae subspecies, 400 spores per gram (as indicated by the manufacturer.
Potassium(K)	231.4 mg kg^−1^	
Calcium(Ca)	564 mg kg^−1^	- The subspecies included *Glomus mosseae, Glomus intraradices, Glomus etunicatum,* and *Scutellospora dipurpurescens*.
Magnesium (Mg)	147.6 mg kg^−1^	- Zn was applied at sowing at a depth of 5 cm (40 kg ha^−1^)

**Table 2:** Application effect of Zinc fertilizer and mycorrhizal inoculation on yield attributes of bread wheat.

	SDW (g)	SL (cm)	RDW (g)	RL (cm)	Spike Number per Plant	Grain Number per Spike	Grain Weight per Spike	1000 Grain Weight (g)
**Control**	0.30 ±0.01 ^cd^	36.48 ±1.05 ^b^	0.29 ±2.13 ^c^	30.65 ±0.35 ^e^	1.75 ± 0.03 ^b^	35.43 ±0.76 ^b^	1.78 ±0.08 ^a^	46.19 ±0.32 ^a^
**Zn**	0.43 ± 0.20 ^b^	38.09 ± 0.54 ^ab^	0.39 ± 0.09 ^bc^	32.48 ± 0.08 ^d^	1.50 ± 1.25 ^bc^	34.52 ± 0.01 ^c^	1.59 ± 0.54 ^b^	51.07 ± 1.65 ^b^
**AMF**	0.48 ± 0.20 ^ab^	39.64 ± 0.32 ^ab^	0.42 ± 0.50 ^b^	38.52 ± 1.76 ^b^	1.75 ± 0.87 ^b^	35.19 ± 0.90 ^b^	0.34 ± 0.01 ^d^	46.38 ± 1.20 ^b^
**AMF+Zn**	0.54 ± 0.45 ^a^	42.71 ± 0.75 ^a^	0.54 ± 1.65 ^a^	42.06 ± 2.00 ^a^	2.00 ± 0.09 ^a^	36.10 ± 0.87 ^b^	1.77 ± 0.70 ^a^	47.68 ± 2.87 ^ab^
**Drought**	0.26 ± 0.43 ^d^	31.99 ± 1.43 ^c^	0.26 ± 0.07 ^c^	29.05 ± 0.98 ^f^	1.00 ± 0.06 ^c^	38.83 ± 0.39 ^a^	1.83 ± 0.90 ^a^	33.86 ± 1.33 ^c^
**Zn+Drought**	0.28 ± 0.97	32.15 ± 0.98	0.28 ± 0.01	29.99 ± 1.09	1.23 ± 0.09	36.90 ± 0.01	1.85 ± 0.99	44.65 ± 0.13
**AMF+Drougt**	0.37 ± 1.45	35.09 ± 0.09	0.33 ± 0.12	35.08 ± 0.23	1.44 ± 1.34	37.21 ± 1.23	0.98 ± 1.06	43.17 ± 0.05
**AMF+Zn+Drought**	0.39 ± 0.04 ^c^	33.10 ± 0.3 ^c^	0.37 ± 2.54 ^bc^	36.95 ± 1.00 ^c^	1.75 ± 0.12 ^b^	38.78 ± 0.56 ^a^	0.99 ± 1.03 ^c^	40.62 ± 1.05 ^bc^

Grown under water-stress conditions, SDW = Shoot dry weight, SL = shoot length, RDW = root dry weight, RL = root length. Values in columns followed by different letters are significantly different at *p* ≤ 0.05. Means ± standard deviation.

## Data Availability

The data presented in this study are available on request from the corresponding author. The data are not publicly available due to privacy and ethical reasons.
